# The societal role of meat—what the science says

**DOI:** 10.1093/af/vfac098

**Published:** 2023-04-15

**Authors:** Peer Ederer, Frédéric Leroy

**Affiliations:** GOALSciences at Global Food and Agriculture Network, Rapperswil, Switzerland; Industrial Microbiology and Food Biotechnology (IMDO), Faculty of Sciences and Bioengineering Sciences, Vrije Universiteit Brussel, Brussels, Belgium

**Keywords:** animal source foods, meat science, nutrition, sustainable livestock, economics of protein, ethics of livestock

Eating meat has been the aspiration for an enjoyable and nutritious meal in most cultures and during most times for at least as long as there are written records, and likely far back to the earliest days of our genus some 2 million yr ago. Nonetheless, history also indicates that there has been frequent and prominent advice to abstain from meat or even prohibit its consumption, for cultural, spiritual, nutritional, or economic reasons. The societal debate around the value of meat is neither new nor has it been dispassionate. Science has been a participant in this debate from early on as well. While Pythagorean communities abstained from meat based on reincarnation theories, Aristotle came to the reasoned conclusion based on everything that he knew about 2,300 yr ago: “*The tame animals are for the use and nourishment of mankind, while the wild ones, if not all, most of them, are on account of nourishment and help, in order that clothes and other tools come to be from these. And therefore, if nature does nothing in vain or without a purpose, it is necessary that nature made all of these on account of humans*” (Aristotle, Politics, 1256b10-22). It is therefore fair for every generation to reask this question considering the best and most recent scientific evidence available: should eating meat in sufficient portions be a common and important part of the standard human diet?

This Special Issue of Animal Frontiers aims to provide a synopsis of answers which represent the currently available best scientific evidence. The answers are given on major considerations pertaining to eating meat, including its impact on human nutrition and health, environmental sustainability, economic affordability, and ethical justification. To this end, we invited a broad group of leading international scientists to interpret the scientific evidence for the benefit of making it accessible to the communities of policy makers, industry practitioners, journalists, common consumers, and fellow scientists alike. Our request of the authors was not to reflect on the most granular levels of current scientific argumentation on each of these topics. That would have been impossible and would never do justice to the quality and intensity of these debates within the scientific community. Instead, we asked them to derive what can be robustly learned and has most societal significance, from the scientific evidence as it currently stands.

As guest editors of this Special Issue, we wish to emphasize our trust in the value of scientific debate, and in the ongoing questioning and challenging of what may appear as common knowledge or as an established paradigm. Science progresses by asking questions more so than by providing answers. We take Karl Popper’s epistemology as a guide, so that at best, we can know what is not true. Similar principles characterize this Special Issue: we appreciate and ask for debate on how to interpret the scientific evidence; we decidedly reject torturing the data until it confesses to a desired outcome; we want to neither suppress the inherent complexity of the subject; nor do we want to hide behind it.

## Livestock and Human Health

As it is often argued that the eating of meat is justified by its contribution to the nutritional needs of global populations, we felt that this was the first key element that needed to be confronted with scientific evidence. The opening article of this Special Issue, “The role of meat in the human diet: Evolutionary aspects and nutritional value” (Leroy et al., 2023), initiates the discussion with the following questions: 1) is meat indeed to be considered as a meaningful part of the species-adapted diet of humans; 2) are there nutrients that can become compromised when abstaining from meat; 3) how does meat contribute to the supply of these nutrients globally; and 4) which risks may be created by a large reduction in meat consumption? The article demonstrates that *Homo sapiens* evolved to be persistent and frequent meat eaters, so that it can be assumed that meat is at least compatible with human anatomy and metabolism. Moreover, given that meat represents a high-quality food matrix for digestibility and absorption of a broad spectrum of nutrients, several of which being already limiting factors in diets worldwide, it seems fair to state that the dietary role of meat is not straightforward to replace. In fact, populations that have scant access to meat tend to suffer from the typically expected health problems associated with low intake of specific micronutrients found in meat, or from deficient quality protein intakes. To sum up, the regular consumption of meat appears to bestow multiple and important nutritional benefits.

Whereas the above-mentioned arguments speak in favor of *some* meat consumption, they tell us little about optimal or maximum intake levels. The corresponding question to the health benefits is whether there are also health risks in eating meat (in particular with respect to red and processed meats and their impact on noncommunicable diseases), and at what dosages such risks may be incurred. Experts in this domain took up the challenge in their article “Non-communicable disease risk associated with red and processed meat consumption – Magnitude, certainty, and contextuality of risk” (Johnston et al., 2023). Based on GRADE methodology, the international standard for evidence-based health recommendations, it was concluded that causality assumptions are of low to very-low certainty only. Claims for further meat restriction below the current intake levels are mostly made based on associative correlations obtained from some observational studies, which suffer from potential bias and residual confounding. Such claims are quite open to interpretation and do not seem sufficient to merit strong public policy action. Even if such risks materialize, they would be trivial (from an absolute risk perspective) and depend on interindividual differences, preparation methods, and the quality of the background diet. Because of the heterogeneity in types and degree of processing and the potential concomitant formation of harmful compounds, precaution may be warranted for the intake of processed meats beyond reasonable levels. Taken together, reducing meat consumption may backfire as it could further undermine nutrient security, especially in populations with elevated needs.

## Livestock and the Environment

Apart from the individual health situation, humanity also faces a collective risk. The human species has become so pervasive on Earth, that its activities may be harming biodiversity and the capacity of natural resource cycles (e.g., water, carbon, phosphorus, and nitrogen) to maintain themselves within stable limits. Being mediated naturally, to some extent, via the large resource pools of oceans, atmosphere, natural biosphere, and land surface, these cycles could strongly alter other biological cycles, possibly resulting in rapid climate change or other natural phenomena. Livestock agriculture has its role to play: the animals from which meat (as well as dairy, eggs, hides, manure, etc.) is gained are not only numerous by themselves, but also consume significant amounts of agricultural resources. A crucial question then is whether these livestock systems consume more resources than sustainable circulatory ecosystems can afford.

To address this, we asked specialists in the domains of agriculture and (agro-)ecology to provide us with their perspective in their article “Ecosystem management using livestock: embracing diversity and respecting ecological principles” (Thompson et al., 2023). Ultimately, the answer depends on what is the desired end state of an ecosystem. If the aspiration is to return to a state of Nature nearly untouched by *Homo*, this must be dismissed as illusionary, arbitrary, and arguably impossible. The latter is not only because it is far beyond human technological means, but also because human impact has altered the Earth already so much, that the clock cannot be turned back (assuming there was even a consensus to which time it should be returned: 500, 5,000, or 50,000 yr back). More realistically, an end state should be sought in which the resource cycles can be reasonably stabilized and where today’s remaining biodiversity can be sustained and ideally improved. Such an endeavor will most likely have to include very large tracts of savannah-type landscapes in the temperate climate zone latitudes, neither forested nor deserted, as these were the default setting in which much of today’s biosphere evolved (including *Homo sapiens*). These landscapes cannot be reinstated without large-scale intervention by (human-managed) ruminant herds. Another argument for the role of animals, both ruminants and monogastrics, is that they are essential to optimize and valorize crop agriculture in food-generating ecosystems. Even if feed–food competition needs to be further mitigated and balanced according to nutritional requirements globally, plant-based production does not only lead to human-edible food, but also large amounts of inedible biomass. Livestock are the most likely viable option to return the nutrients captured in this biomass back into the natural cycle, while producing high-quality human-edible food. Moreover, the amount of crops and the surface they need would have to expand to compensate for reductions in animal-sourced food (and the highly bioavailable nutrients it contains). The outcome of unintended economic, social, and environmental consequences when abandoning livestock could prove catastrophic to the already shaky ecological balance of the resource cycles and the remaining natural capital. In short, human-managed livestock systems must be part of the solution to environmental sustainability.

If the above scenario based on the sustainable integration of animal and plant agricultural is a possible desired end state, this next question follows: how much of what type of agriculture and livestock system needs to be practiced where, to achieve optimal land use and a sustainable food system? Further: would such a scenario be capable of producing enough meat to satisfy the potential demand which a global population of soon enough 10 billion people would want to eat, given the nutritional benefits described above? We consulted experts in the more quantitative aspects of food system transformation, and they do not think that this answer can readily be given. In their article “Challenges for the fair attribution of livestock’s environmental impacts: the art of conveying simple messages on complex realities” (Manzano et al., 2023), it is shown that our understanding of the critical resource cycles and pools is still too underdeveloped to estimate the sustainable carrying capacity of the Earth for livestock of all the various species. What we do know with certainty is that the accounting systems that are currently used to describe the impact of livestock systems on the resource cycles have important limitations. This is not necessarily problematic, if these limitations are well acknowledged and reductionist tunnel vision is avoided. At the same time, we should also strive to develop and operationalize better metrics where feasible. This disclaimer applies to all aspects of the natural resource cycle, for instance where the impacts of ruminant’s methane emission on the carbon cycle in the atmosphere and the soils are estimated, where nitrogen cycles are measured or where the water cycles are being evaluated. Little good can be expected from such impact estimations if the accounting systems are not updated to the current state-of-the art knowledge, and if important gaps in empirical knowledge are not swiftly filled with committed research efforts. To achieve the holy grail of holistic, transparent, and fair metrics, scientists from a variety of disciplines and representing a broad range of views and skills need to work together.

## Livestock and Socioeconomics

The first four articles in this special issue cover the basic requirements for ensuring human nutritional and agro-ecosystem health. Meeting these basic requirements will require coordinated effort in the food system value chains and greater capital investments. In 2017, the cheapest price for a basket of food items, including animal-sourced foods, that satisfy the minimum nutrition required by an individual, was around three purchasing power parity adjusted U.S. dollars per person per day in most countries, an amount not affordable to about 40% of the global population. Due to the COVID-19 pandemic, the Ukraine crisis, and strong inflationary forces, this percentage is likely to have increased by 2022. The article “Affordability of meat for global consumers and the need to sustain investment capacity for livestock farmers” (Ederer et al., 2023) maintains that all the long-term health and productivity harms that undernourishment causes, is not only an avoidable human tragedy but also a huge loss in economic opportunity. Expanding animal production output is the most readily available way to nourish the world sufficiently in the future. To achieve this, today’s livestock production processes must become more efficient, leading to more affordable consumer prices of meat, milk, and eggs, which would be a key contribution to making sufficiently nutritious food universally available. Not the only, but one of the key necessary conditions for such a future will be large investments to build livestock food systems that are environmentally sustainable as well as nutritionally adequate. With examples of the judicious policies and widespread adoption of innovative livestock interventions reported on in this issue, farmers, herders, agribusiness, and policy makers shall be inspired that this is feasible.

More animals, being produced more cost-effectively, may create ethical challenges for livestock keeping, both during their life in terms of animal welfare, and in the inevitability of their death to supply meat. We therefore must consider the ethical dimensions as well. In their article “Is meat eating morally defensible? Contemporary ethical considerations,” Croney and Swanson (2023) deliberate that the case either for or against meat on ethical grounds purely from the perspective of the animal is weak. Ethicists have been impaling themselves in their debates on philosophical principles for decades without much useful outcome. However, careful ethical analysis shows that if meat is economically required to provide for human health, and as long as substantial portions of the global population cannot access sufficient amounts of meat, then this shortfall has ethical primacy over considerations of the conditions of the animals. As long as there are human mouths to feed with meat and no better alternative in sight, then humans enjoy clear ethical priority over the animal.

Is there an alternative looming on the horizon? Over a billion dollars are being invested in creating technologies based on cell culture that promise to be able 1 d to produce a food product equivalent to meat, but without the need to slaughter an animal. The technology is to grow animal cells in a bioreactor, aiming to achieve a similar biological and nutritional outcome as in traditional meat. Doing so at the required scale would obviate the need to grow, feed, and slaughter animals. The promise is also that the environmental burden of its production would be lower than the burden of livestock production. If the assumption is correct that this process will be more cost-effective and have less-environmental impact than today’s livestock systems, then the global nutrient gap could eventually be closed in this way. These assumptions are currently far from realistic, argue Wood et al. (2023) in their article “‘Cellular agriculture’: Current gaps between facts and claims regarding ‘cell-based meat.’” The relevant technologies are not new, and their history of several decades of research as well as remaining technological issues suggests it may take considerable time to overcome the hurdles while it is unclear that the cost can be brought down sufficiently to become a viable economic, nutritional, or environmental alternative to farming animals.

Does all this mean that the meat sector requires no changes, and that the global livestock and meat industry may continue as it has all along? That is most unlikely. The twin challenges of closing the global nutrient gap and achieving environmental sustainability are large. More research is needed than ever before, in all aspects of the sciences. This requires dedicated effort across disciplines, and between the sectors of private industry, public policy, governments, and scientific organizations. The Special Issue ends with the article titled: “Challenges and opportunities for defining the role and value of meat in our global society and economy” (Polkinghorne et al., 2023) highlighting examples of success of how more knowledge can be generated faster, and thus deliver much-needed better solutions.


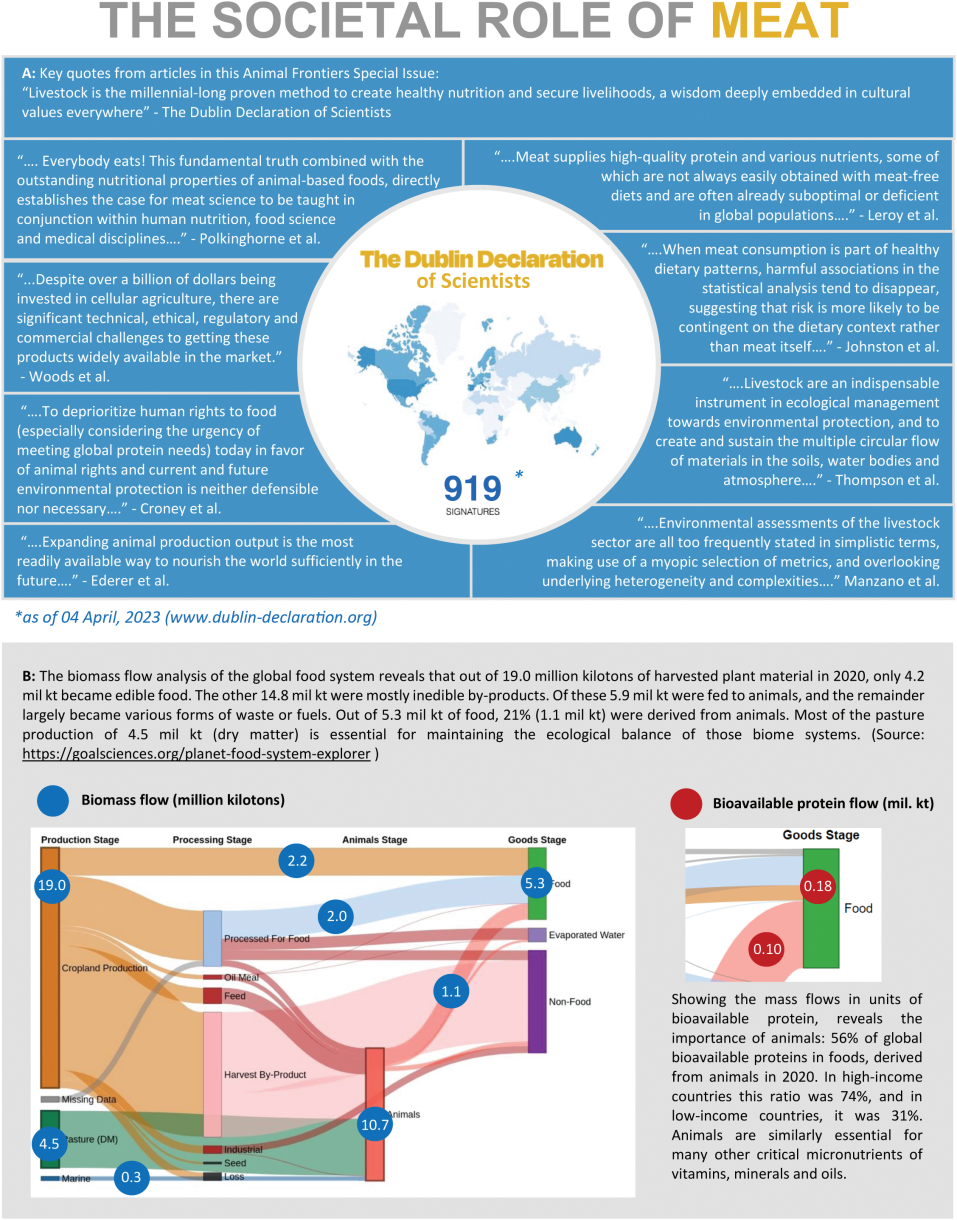


## The Dublin Declaration of Scientists

As part of our overall effort, we formulated The Dublin Declaration of Scientists (The Dublin Declaration of Scientists on the Societal Role of Livestock, 2023). We invite all scientists from around the world to support the Declaration by signing it digitally, and thus give our science a voice that too often is silenced. Instructions for the signature can be found at www.dublin-declaration.org. The last paragraph of the Dublin Declaration was taken from the text of the 2021 UN Food System Summit final documentation on Sustainable Livestock, which we believe is a most appropriate statement to conclude this editorial piece. It reads: “*Human civilization has been built on livestock from initiating the bronze-age more than 5000 years ago toward being the bedrock of food security for modern societies today. Livestock is the millennial-long proven method to create healthy nutrition and secure livelihoods, a wisdom deeply embedded in cultural values everywhere. Sustainable livestock will also provide solutions for the additional challenge of today, to stay within the safe operating zone of planet Earth’s boundaries, the only Earth we have.*”

## About the Author(s)

**Peer Ederer** is founder and director of GOALSciences, the Global Observatory of Accurate Livestock Sciences, which has the mission to research and communicate scientific evidence about the role of animals in the global food system. He has an MBA degree from Harvard University, a PhD in financial economics and holds an adjunct professorship for innovation studies. He has extensive experience in strategy advisory to private companies and public bodies in the global food system and is a frequent presenter on related topics. He has been engaged in scientific research in cooperation with globally leading universities from around the world and is a member of the Scientific Council of the World Farmers Organisation.

**Frédéric Leroy** graduated as a Bioengineer (Ghent University, 1998) and obtained a PhD in Applied Biological Sciences at the Vrije Universiteit Brussel (VUB, 2002), where he now holds a professorship in food science and (bio)technology. His research deals with food processing, human and animal health, and interdisciplinary food studies. He is a Board member of various academic nonprofit societies, that is, the Belgian Association of Meat Science and Technology (president), Belgian Society for Food Microbiology (president), and Belgian Nutrition Society. On a nonremunerated basis, he also serves on various Scientific Boards (e.g., the World Farmers’ Organization and the FAO/COAG Sub-Committee on Livestock).
